# Fast evaluation technique for the shear viscosity and ionic conductivity of electrolyte solutions

**DOI:** 10.1038/s41598-022-10704-z

**Published:** 2022-05-04

**Authors:** Takeshi Baba, Seiji Kajita, Tohru Shiga, Nobuko Ohba

**Affiliations:** Toyota Central R&D Labs., Inc., 41-1, Yokomichi, Nagakute, Aichi 480-1192 Japan

**Keywords:** Computational methods, Molecular dynamics, Batteries

## Abstract

With the growing need to obtain ideal materials for various applications, there is an increasing interest in computational methods to rapidly and accurately search for materials. Molecular dynamics simulation is one of the successful methods used to investigate liquid electrolytes with high transport properties applied in lithium-ion batteries. However, further reduction in computational cost is required to find a novel material with the desired properties from a large number of combinations. In this study, we demonstrate an effective fast evaluation technique for shear viscosity and ionic conductivity by molecular dynamics simulation for an exhaustive search of electrolyte materials with high transport properties. The proposed model was combined with a short-time correlation function of the stress tensor and empirical relationships to address the issues of inefficient and uncertain evaluation by conventional molecular dynamics methods. Because we focus on liquid electrolytes consisting of organic solvents and lithium salts, our model requires dissociation ratio and effective diffusion size of lithium salts. Our method is applied to search for the compositional combinations of electrolytes with superior transport properties even at low temperatures. These results correlate well with experimental results.

## Introduction

There has been an increasing interest in the development of energy storage materials, as their application expands to include electric vehicles and renewable energy storage. Rechargeable lithium-ion batteries (LiBs) are one of the most common power sources and are still developed for conveniences, such as high energy density and rapid charging, as well as for safety^1^. When LiBs are discharged, the following reactions occur on the cathode (positive electrode; typically lithium transition metal oxides, such as LiCoO_2_) and anode (negative electrode; typically graphite):

1 $$\begin{array}{*{20}c} {{\text{LiCoO}}_{{2}} \to {\text{Li}}_{{{1 - }x}} {\text{CoO}}_{{2}} + x{\text{Li}}^{ + } + xe^{ - } ,} \\ \end{array}$$

2 $$\begin{array}{*{20}c} {x{\text{Li}}^{ + } + x{\text{e}}^{ - } + x{\text{C}}_{{6}} \to x{\text{LiC}}_{{6}} .} \\ \end{array}$$

During charging, the reactions are reversed. During discharging/charging, while electrons flow through the circuit between the anode and cathode, lithium ions flow through the electrolyte. The electrolyte is one of the key factors that determine the performance of the LiBs because it must satisfy various requirements, such as electrochemical stability with respect to electrodes, chemical stability over a wide temperature range, safe materials (i.e., nonflammable/nonexplosive), and good ion transportability^2^. While various types of electrolytes have been studied, including ionic liquids, polymers, and inorganic solids, the most commonly used is organic liquids dissolved with lithium salt as the support^1,2^. Carbonates are typical organic liquids because they are good solvents for supporting salts; they accelerate conductivity and have good electrochemical and chemical stability. However, a real battery system is considerably complex, and the role of the electrolyte is not limited to carrier transport. For example, when a graphite anode is used, many organic liquids undergo reductive decomposition, creating solid-electrolyte interphase (SEI) films on the anode. Since they have a significant impact on the performance of LiBs, multicomponent electrolytes with additives, for example, have been developed to produce better SEI films^1^. Moreover, some organics are flammable and have modest ion transportability at low temperatures. To overcome these disadvantages and enhance the performance of the LiBs, new electrolytes with high salt concentrations, low driving temperatures, and flame resistance have been proposed recently^3–5^.

Experimental approaches based on trial and error, experience, and intuition have been frequently used in the research on appropriate materials. This limits the possibility of identifying novel materials outside the scope of experience. For electrolytes, the bottleneck in their material research is a large number of material combinations, because electrolyte materials are composed of multiple solvents and supporting salts with varying material types and compositional ratios. Therefore, the material informatics (MI) techniques, which apply the recently developed machine learning technology, are expected to be useful for material development^6,7^.

MI mainly consists of "direct problems," in which the properties of a material are predicted from basic information, such as molecular structure and composition, and "inverse problems," in which the structure and composition are determined from the target properties. It is necessary to solve the inverse problems in material design. However, there is a limited number of studies using this technique. In a previous study^8^, we have proposed a method to search for the optimal molecular structure by recursively performing the direct and inverse problems. Specifically, a structure generation algorithm based on the Monte-Carlo tree search method has been prepared and property prediction has been performed for the generated molecules. Subsequently, the results are fed back to the structure generation algorithm to update the algorithm and generate molecules that are expected to have improved properties; this process is repeated. Since this is an iterative optimization method, speed and accuracy are important factors for structure and property prediction.

In cases where speed is required for property prediction, an evaluation method for properties based on model simulation is more advantageous. The advanced electrolyte model proposed by Gering^9^ is well known for predicting the physical properties of an electrolyte, such as ionic conductivity and shear viscosity. Although the prediction accuracy is high, it is difficult to apply this model to search for novel materials because the parameters used must be determined experimentally in advance. In contrast, molecular simulation techniques, such as first-principles calculations and molecular dynamics (MD), can predict properties of even novel molecules, because these schemes need only coordinates of molecular configurations as the inputs. However, these techniques are limited by their speed. The novel oil molecular design by Kajita et al.^8^ has exhibited a similar problem because the viscosity was used as the target property. Nonetheless, they have succeeded in achieving both prediction accuracy and speed by introducing an empirical physical model while capturing the material features from short MD simulations.

In this study, based on the development of viscosity prediction techniques for oil molecules, we demonstrate the effectiveness of a fast prediction technique for viscosity and ionic conductivity for a large-volume search for electrolytes used in LiBs and capacitors. While the original model is generally applicable to viscosity of single liquids, we extend the model to predict conductivity of complex liquids that consist of organic solvents and lithium supporting salts. Namely, information of lithium salts is included in the model. Further, the predicted results are compared with the experimental data to confirm their accuracy and variability.

## Results and discussion

### Fast evaluation technique for transport properties

Conventionally, the shear viscosity and ionic conductivity of a liquid can be estimated using equilibrium MD (EMD) simulation^10^, as mentioned in the Methods section. To avoid the uncertainty associated with the time-ensemble averaging characteristics of conventional MD-based transport property calculations, a previous study^8^ considered the Arrhenius relation with an additional quadratic term^11^ between the viscosity $\eta$ of a liquid and its shear modulus $G_{\infty }$:

3 $$\begin{array}{*{20}c} {\log \eta = \lambda_{2} \left( {\frac{{G_{\infty } }}{T}} \right)^{2} + \lambda_{1} \frac{{G_{\infty } }}{T} + \lambda_{0} ,} \\ \end{array}$$

where $\lambda_{0} , \lambda_{1} {\text{, and }}\lambda_{2}$ are parameters, $T$ is the temperature, and $G_{\infty }$ is proportional to the shear modulus $\overline{\Phi }$.

4 $$\begin{array}{*{20}c} {\overline{\Phi } = \mathop {\lim }\limits_{t \to 0} \frac{V}{{k_{B} T}}\frac{d}{dt}\mathop \smallint \limits_{0}^{t} dt^{\prime}\langle P_{\alpha \beta } \left( {t^{\prime}} \right)P_{\alpha \beta } \left( 0 \right)\rangle, } \\ \end{array}$$

where *k*_*B*_ is the Boltzmann constant,* V* is the volume, and $P_{\alpha \beta }$ is the off-diagonal element of the stress tensor in the system (Eq. 13). Here, we use the following approximation of Eq. (4):

5 $$\begin{array}{*{20}c} {\overline{\Phi } \simeq \frac{V}{{k_{B} T\delta t}}\mathop \smallint \limits_{0}^{\delta t} dt\langle P_{\alpha \beta } \left( t \right)P_{\alpha \beta } \left( 0 \right)\rangle, } \\ \end{array}$$

where $\delta t$ is the short time for the order of molecular vibrations. As discussed in a previous paper^8^, the estimation of $\overline{\Phi }$ in Eq. (5) can be evaluated by a few samplings because the quantity is related to the $\delta t$ correlation. Therefore, using the relationship of $G_{\infty } \propto \overline{\Phi }$ and Eq. (3), the viscosity can be efficiently evaluated. Additionally, to improve the viscosity evaluation, we used the empirical van Velzen model^12,13^, in which the logarithm of liquid viscosity has a linear relationship with the reciprocal of the absolute temperature to the boiling point $T_{b}$ of the system. The prediction expression for viscosity is as follows:

6 $$\begin{array}{*{20}c} {\log \eta = A\overline{\Phi }^{2} \left( {\frac{1}{T} - \frac{1}{{T_{b} }}} \right)^{2} + B\overline{\Phi }\left( {\frac{1}{T} - \frac{1}{{T_{b} }}} \right) + \log \eta_{0} ,} \\ \end{array}$$

where *A*,* B*, and $\eta_{0}$ are the model parameters. The values were determined by fitting Eq. (6) using the calculated shear modulus of the reference molecules, the experimental values of viscosities and boiling points. The proposed fast evaluation method is typically applied to liquid systems because the viscosity and boiling point in Eq. (6) are unique concepts for liquids.

For conductivity prediction, the Walden rule^14^, in which the molar conductivity $\Lambda$ is inversely proportional to the viscosity, was implemented:

7 $$\begin{array}{*{20}c} {\Lambda \propto \left( {\frac{1}{\eta }} \right)^{s} ,} \\ \end{array}$$

where $s$ is a fractional parameter that represents the deviation from the ideal Walden relation^15^. The Walden relation is often interpreted using the Stokes–Einstein (S–E) relation:

8 $$\begin{array}{*{20}c} {D_{i} = \frac{{k_{B} T}}{{l\eta^{s} R_{i} }},} \\ \end{array}$$

where $D_{i}$ is the self-diffusion coefficient of the carrier ions, *i* is the ion species, and $l$ is a parameter determined by the relationship between the diffusing particle and solvent size, which is $4\pi$ for a slip condition and $6\pi$ for a stick condition in an ideal S–E relationship (*s* = 1), and $R_{i}$ is the diffusion particle radius. For simplicity, we used the Nernst–Einstein (N–E) relation (Eq. 15) and assumed a monovalent 1:1 salt, i.e., *i* =  + and – for cations and anions, respectively; the absolute value of the ion charge ($z_{i}$) was one $\left( {\left| {z_{ + } } \right| = \left| {z_{ - } } \right| = 1} \right)$, and the number of cations and anions were the same ($N_{ + } = N_{ - }$). Therefore, the ionic conductivity $\sigma$ became

9 $$\begin{array}{*{20}c} {\sigma = \frac{{Ne^{2} }}{{Vl\eta^{s} }}\left( {\frac{1}{{R_{ + } }} + \frac{1}{{R_{ - } }}} \right) = Cc\left( {\frac{1}{\eta }} \right)^{s} \Gamma ,} \\ \end{array}$$

where $e$ is the elementary electric charge, *N* is the number of ions, $C = \frac{{e^{2} }}{l}$, *c* is the carrier concentration ($c = \frac{N}{V}$), and the terms $\left( {\frac{1}{{R_{ + } }} + \frac{1}{{R_{ - } }}} \right)$ are consolidated as $\Gamma$. If $l$ and $\Gamma$ are constant, $\Lambda = \frac{\sigma }{c}$, which is equivalent to Eq. (7). Notably, although the temperature term does not appear explicitly in Eqs. (7) and (9), it is included indirectly because the viscosity depends on the temperature.

The value of $\Gamma$ was determined by the degree of pairing and the size of the ion species. To obtain $\Gamma$ in a real system, the correlations between ion species must be calculated using the Einstein relation of conductivity $\sigma_{E}$ (Eq. 16), which is undesirable in terms of computational cost. Borodin et al. compared the values estimated by $\frac{{\sigma_{E} }}{{\sigma_{NE} }}$ (Eqs. 15 and 16) with the degree of ion dissociation $\alpha$ estimated from the structural information ($1$ for full dissociation and $0$ for full association) in an MD simulation of a system consisting of ethylene carbonate (EC) and lithium bis((trifluoromethyl)sulfonyl)amide (LiTFSA)^16^ or EC–LiPF_6_^17^. Although $\alpha$ was underestimated, the qualitative trends were consistent. Therefore, we determined the $\alpha$ using the structure information obtained by MD simulation, which can be obtained at a low cost, and prepared a functional form including $\alpha$ for $\Gamma$. Similar to the viscosity model, parameters *C* and *s* were determined by fitting the reference data.

### Validation of the model

Parameters *A*,* B*, and *η*_0_ of the viscosity model in Eq. (6) were determined by fitting the experimental values of the shear viscosity and $\overline{\Phi }$ obtained from the MD calculations. We used 193 materials, including 43 typical organic solvents, 138 electrolytes (see Supplementary Information for details), and 12 oil molecules used in a previous study^8^ (at 40 °C only), to cover a wide range of viscosities. $\overline{\Phi }$ is the short-time integral of the shear stress–time correlation function, and $\delta t$ was set to 10 fs.

The boiling point was included in the viscosity model. The boiling points of all targeted organic solvents were known, while those of the oil molecules and electrolyte solutions have not been reported to the best of our knowledge. Thus, the boiling point of the oil molecules was estimated using the group contribution method (the Joback method)^18^. Using the boiling point $T_{b,i}$ of the constituent solutes, solvents $i,$ and mole fraction $x_{i}$, the boiling point $T_{{b,{\text{sol}}}}$ of the electrolyte was determined to be

10 $$\begin{array}{*{20}c} {T_{{b,{\text{sol}}}} = \mathop \sum \limits_{i} x_{i} T_{b,i} .} \\ \end{array}$$

The boiling points of supporting salts, such as LiPF_6_, have not been reported because they thermally decompose with increasing temperature; only their melting points have been reported. Therefore, a regression equation for the melting and boiling points was created using the lithium salt, in which the melting and boiling points were known, and the boiling point was estimated from the melting point of the supporting salt (see Supplementary Information for the specific method).

**Figure 1:**
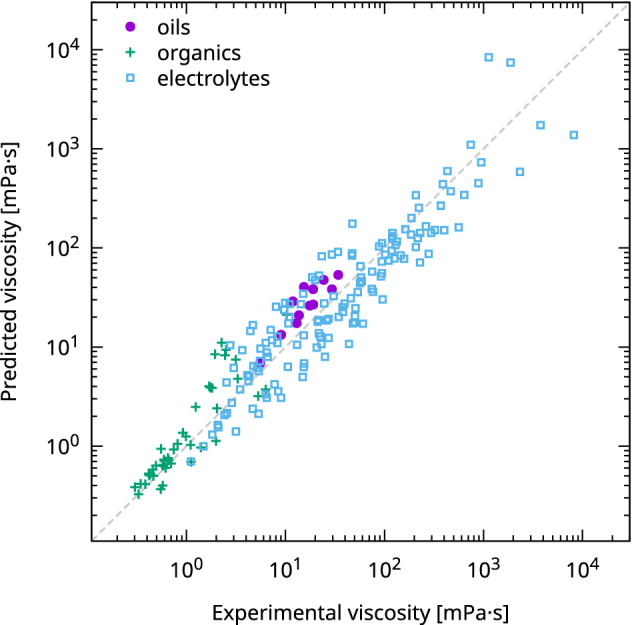
Comparison of the predicted and experimental shear viscosity (in mPa·s). The markers represent a kind of solvent.

The values of *A*,* B*, and *η*_0_ were calculated as 5.017 × 10^6^, 3.787 × 10^3^, and 0.311 mPa·s, respectively. The predicted and experimental viscosities obtained using these parameters are shown in Fig. 1. The root mean square error (RMSE) between the experimental and predicted values of the logarithmic viscosity was 0.28, indicating that a good model was constructed regardless of the liquid used.

The ionic conductivity model of Eq. (9) was fitted to 203 electrolytes comprising various solvents containing LiPF_6_, lithium bis(fluorosulfonyl)amide (LiFSA), and LiTFSA as the support salts (see Supplementary Information). Since the $c$ of some electrolytes has not been reported, $c$ and $\alpha$ estimated from the MD simulation results were used. For $\Gamma$, the following equation was used:

11 $$\begin{array}{*{20}c} {\Gamma = \frac{1}{{a_{s} \left( {1 - \alpha } \right) + a_{i} \alpha }}.} \\ \end{array}$$

This function became $\frac{1}{{a_{i} }}$ when $\alpha \to 1$ and $\frac{1}{{a_{s} }}$ when $\alpha \to 0$ because the target electrolyte was only a Li-supported salt, i.e., the cations were only lithium ions. Conventionally, the radius of lithium was sufficiently smaller than that of the anion; therefore, fully dissociated, $\frac{1}{{{\text{R}}_{ + } }}$ became dominant over $\Gamma$. However, a relatively large diffusing particle size can be assumed when lithium ions aggregate. Therefore, the ionic radius of lithium (0.76 Å) was used for $a_{i}$, and the values using the molecular volumes estimated by the first-principles calculations were used for $a_{s}$, where the values of LiPF_6_, LiFSA, and LiTFSA were 3.06, 3.64, and 4.15 Å, respectively. Notably, a similar concept regarding the effective solute radius of lithium in electrolytes was discussed by Berhaut et al.^19^ They determined the Jones–Dole–Kaminski radii using the relative viscosity of the solutions and model fitting. They found that those of EC–dimethyl carbonate (DMC) (50:50 wt%)–LiPF_6_ (1 mol/L) and EC–DMC (50:50 wt%)–LiFSA (1 mol/L) varied from 4.1 to 4.7 and 4.9 to 4.0 Å, respectively, in the temperature range from 20 to 60 °C. Although it was difficult to make a direct comparison because the effect of the temperature and the ion dissociation ratio was included in their evaluation, the values used in our study were slightly underestimated; however, the trend was consistent.

**Figure 2:**
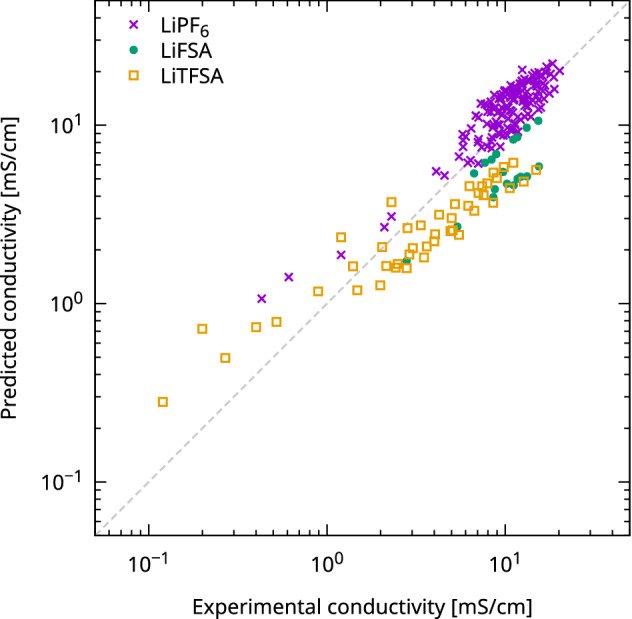
Comparison of the predicted and experimental ionic conductivity (in mS/cm). The markers represent a kind of Li salts.

The conductivity model parameters *C* and *s* were 30.8 and 0.59, respectively. Berhaut et al. reported that the fractional Walden parameters of EC–DMC (50:50 wt%)–LiPF_6_ (1 mol/L) and EC–DMC (50:50 wt%)–LiFSA (1 mol/L) electrolytes were 0.9 and 0.8, respectively^19^; however, our fitting parameter $s$ was less than these values. This can be attributed to the attempt to express various electrolytes, such as TFSA, and a high-concentration system with a single parameter. The relationship between the conductivities predicted using these parameters and the experimental data is shown in Fig. 2. The RMSE was 3.28 mS/cm. Similar to the viscosity results, a simple expression was used to construct a constant model. The variation and error of the predicted conductivity were not sufficiently discussed, and the measurement method of the conductivity using an electric conductivity meter and battery cells differed in the literature.

### Viscosity and ionic conductivity prediction for typical electrolytes

First, we compared the viscosity and ionic conductivity results of a standard EC–EMC (30:70 vol%)–LiPF_6_ (1 mol/L) electrolyte using a conventional MD method and our proposed fast evaluation method. The conventional MD methods for predicting transport properties can be broadly divided into those using EMD and those using non-equilibrium MD (NEMD). As our fast method was based on the framework of the EMD method, we adopted the EMD method as the conventional MD method for comparison. The prediction of viscosity by the Green–Kubo (G–K)-based EMD method involves the numerical problems mentioned above. Therefore, we adopted an improved approach using a traceless-symmetric method that uses the diagonal component of stress and a fitting method with an analytic function for the running integral (see Method section for detail). This method can predict the viscosity of homogeneous systems with the same accuracy and reliability as the NEMD method^20,21^. For ionic conductivity prediction, two methods were implemented in the conventional MD calculations. The first method determined the diffusion coefficient from the slope of the mean square displacement (MSD) of the Li ions and PF_6_ anion, which was converted by the N–E formula. The other method evaluated the positional information of the Li ions and PF_6_ anions using the Einstein (E) formula. Sampling was performed 10 times with different initial structures using an analysis time of 20 ns for the conventional method and 1 ns for the proposed method. The temperature was set at 298 K.

Table 1 summarizes the characteristics of each method. The mean and standard deviations of the samples and experimental values^22^ are shown for comparison in Table 1. The predicted values of the conventional MD method have a large error with respect to the experimental values because of the use of a general AMBER force field (GAFF)^23^, as discussed by Zhang et al.^24^. Since the prediction accuracy can be improved by adjusting the GAFF parameters^24^ or using a polarized force field^16,17^, the variability was discussed.

**Table 1:** Viscosity and ionic conductivity (298 K) of the EC–EMC (30:70 vol%)–1 M LiPF_6_ electrolyte determined by conventional MD and proposed methods.

**Viscosity [mPa·s]**		
conventional MD (G–K)	34.52	(14.4%)
proposed method	4.47	(2.1%)
experimental^22^	3.0	

**Ionic Conductivity [mS/cm]**		
conventional MD (N–E)	0.90	(15.8%)
conventional MD (E)	0.69	(154.9%)
proposed method	8.94	(6.5%)
experimental^22^	9.33	

The values in the parentheses are standard deviations of the samples. Notably, the sampling time lengths for the conventional and current MD methods are 20 and 1 ns, respectively.

*G–K: Green–Kubo, N–E: Nernst–Einstein, E: Einstein.

As shown in Table 1, the viscosity obtained by the G–K method and the conductivity obtained by the N–E method both have variations of approximately 15%. The E method resulted in an increased variation (150%), in line with the common knowledge that it is more difficult to obtain a statistical mean using this method than using the N–E method^16,17^. In contrast, the prediction variability of the proposed method for viscosity and conductivity was significantly small, indicating the advantage of using short-time correlated information, as discussed in our previous paper^8^. Moreover, the prediction accuracy improved, even with the same MD force field parameters, demonstrating the effect of introducing an empirical physical model. In summary, the proposed method can predict the viscosity and conductivity of an electrolyte more accurately than conventional methods with less than half the variance, and the proposed method uses only 1/20 of the sampling steps of the conventional method. This indicates that even if we could improve the prediction accuracy by changing the force field in the conventional methods, we would have to significantly increase the sampling to obtain the same level of variability as the proposed method. Zhang et al.^25^ showed that the viscosity prediction by the G–K method converges after more than 30–40 independent sampling runs, depending on the system.

### Search for low-temperature driving electrolyte

Next, we investigated a low-temperature driving electrolyte. The ions hardly moved at low temperatures; therefore, the estimation of the transport properties using the conventional MD method required a considerably long production run time. The proposed method used only a short-time correlation of the stress tensor, and the computational time did not increase under low-temperature conditions, which is advantageous for searching a wide composition of materials. We considered a system of fluoroethylene carbonate (FEC) solvent and LiFSA salt. The electrolyte solutions were mixed by varying the ratio of acetonitrile (ACN), methyl acetate (MA), or 1,1,2,2-tetrafluoro-1-(2,2,2-trifluoroethoxy) ethane (D2) with DMC as the solvent to ensure that the diffusivity was considered. We chose these materials because we could confirm that they would not freeze at the desired temperature. Further, experimental measurements of the shear viscosity and ionic conductivity of the same electrolytes were performed to verify the calculation results.

**Figure 3:**
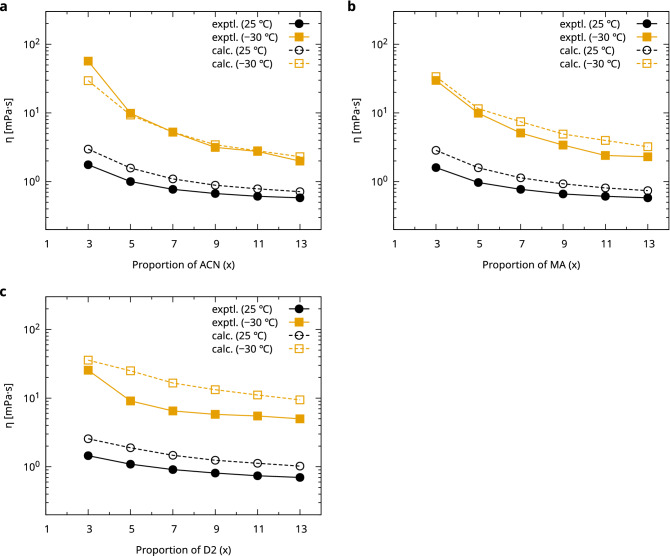
Comparison of the experimental and fast evaluation results of the viscosity for the FEC–X–DMC–LiFSA electrolytes. The molar composition of the electrolyte is FEC:X:DMC:LiFSI = 1:x:y:1. The horizontal axis is the proportion of the X component (x). (**a**) X = ACN (y = 1), (**b**) X = MA (y = 1), and (**c**) X = D2 (y = 3).

Figure 3 shows a comparison of the viscosities at room temperature (298 K) and low temperature (243 K) obtained from the experimental results and the proposed method. Although the proposed method tended toward a slight overestimation, the experimental values and the proposed method followed the same trend. D2 was not included in the material group used for the model fitting of the viscosity; thus, the difference in the experimental and estimated values was larger in this system than in the other systems; however, the trend of the calculated values was in good agreement with the experimental ones.

**Figure 4:**
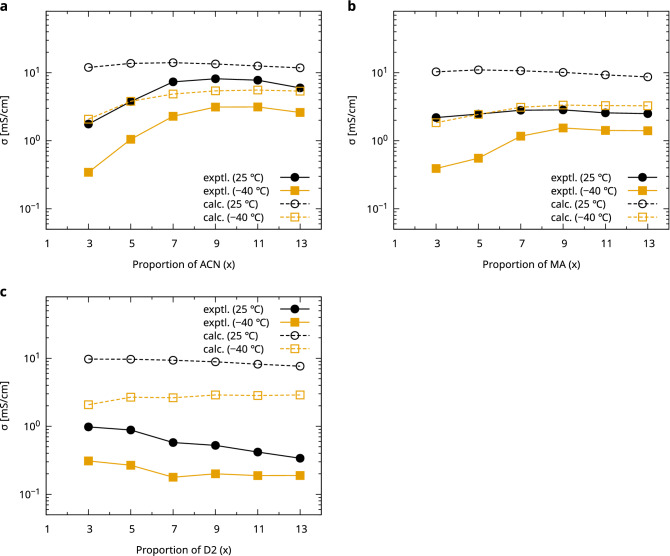
Comparison of the experimental and fast evaluation results of the ionic conductivity for the FEC–X–DMC–LiFSA electrolytes. The molar composition of the electrolyte is FEC:X:DMC:LiFSI = 1:x:y:1. The horizontal axis is the proportion of the X component (x). (**a**) X = ACN (y = 1), (**b**) X = MA (y = 1), and (**c**) X = D2 (y = 3).

Figure 4 shows a comparison between the experimental results and the proposed method for ionic conductivity at room temperature (298 K) and low temperature (233 K). The prediction overestimated the ionic conductivity compared with the experimental values. The conductivity of the FEC–ACN–DMC–LiFSA electrolyte was underestimated by a factor of approximately 3 at both room and low temperatures; however, the tendency of the conductivity for the ACN ratio was similar. Similarly, the experimental trend based on the MA ratio was reproduced for the FEC–MA–DMC–LiFSA electrolyte, although the conductivity was underestimated by a factor of approximately 10 at room temperature. In contrast, the experimental trend of the FEC–D2–DMC–LiFSA electrolyte according to the D2 ratio was not well reproduced, particularly at low temperatures.

The quantitative difference between the experimental measurements and predictions was due to the limitations of the current model representation, particularly in the low-conductivity region. Figure 2 shows that all data below 1 mS/cm were overestimated in the prediction. One possible reason for this is the small amount of reference data used for fitting in this region. Further, the calculation model had a simple assumption of $\Gamma$, as shown in Eq. (11), which did not consider the temperature dependence of the degree of dissociation, depending on the supporting salt and the difference in the size of the ion pairs based on the concentration^19^. Although further studies on quantitative ionic conductivity prediction are required, this result indicates that qualitative features, such as the trend of ionic conductivity with respect to changes in the composite amounts, can be quickly examined in silico.

## Conclusion

We proposed a fast prediction technique for shear viscosity and ionic conductivity, and it is effective for the exhaustive search of the electrolyte solutions with high transport properties used in LiBs and capacitors. The proposed prediction technique combined an extremely short-time correlation function and a physical model, instead of using long-time correlation information that requires high computational costs, for the prediction of transport properties in conventional MD simulations. The fast prediction model for the viscosity was similar to the viscosity prediction model^8^ for oil molecules; however, its accuracy was ensured by expanding the range of materials used for the model fitting. For the electrolytes, this method used only a few tenths of the computational cost and demonstrated good accuracy and variability. Further, the conductivity model used an empirical relationship, using the viscosity obtained at high speed, which eliminated the cost and variability problems of the conventional MD method. Furthermore, the effectiveness of this method was demonstrated by applying it to the search for electrolytes driven by low temperatures and confirming that the experimental values and trends were reproduced. We believe that the proposed method can be used to accelerate MI research.

Unlike viscosity, there was a gap in the quantitative accuracy of the estimated ionic conductivity owing to the complicated physical phenomenon of the conductivity because of the mobility of the solution and the environment of the ion pairs. For example, conductivity was affected by the degree of dissociation of the supporting salts and the diffusion mechanism of the solvated lithium ions, depending on the electrolyte type, concentration of supporting salts, and temperature. Additionally, although our model was based on an electrolyte containing lithium salts, the Walden's relationship used can be applied to materials with ionic conductivity, such as ionic liquids and proton conductors. Since deviations from the ideal Walden's relation are often discussed for these materials, generalizations may be possible from these perspectives. Therefore, there is room for improvement in the model used, and future research is required to improve its applicability and quantitative accuracy.

## Methods

### Conventional MD evaluation for transport properties

The transport properties, such as shear viscosity and ionic conductivity, of a liquid were obtained using an equilibrium MD simulation^10^. The self-diffusion coefficient of the carrier ions was obtained from the MSD slope, and the E or G–K equation was obtained from the velocity autocorrelation function. Theoretically, both equations are equivalent; however, from the viewpoint of numerical accuracy, the latter is generally used for viscosity prediction, while the former is used for conductivity prediction^21^.

To obtain the viscosity $\eta$, the G–K equation was used:

12 $$\begin{array}{*{20}c} {\eta = \mathop {\lim }\limits_{t \to \infty } \frac{V}{{k_{B} T}}\mathop \smallint \limits_{0}^{t} dt^{\prime}\langle P_{\alpha \beta } \left( {t^{\prime}} \right)P_{\alpha \beta } \left( 0 \right)\rangle, } \\ \end{array}$$

where *V*,* T*, and *k*_*B*_ are the volume of the system, the temperature, and the Boltzmann constant, respectively. $\langle \cdots \rangle$ indicates the ensemble average over the multiple time origin (*t* = 0), and $P_{\alpha \beta }$ is the off-diagonal element of the stress tensor, which is

13 $$\begin{array}{*{20}c} {P_{\alpha \beta } = \frac{1}{V}\left( {\mathop \sum \limits_{i}^{N} m_{i} v_{\alpha ,i} v_{\beta ,i} + \mathop \sum \limits_{i > j}^{N} r_{\alpha ,ij} f_{\beta ,ij} } \right),} \\ \end{array}$$

where $m_{i}$ is the mass of the $i$ th particle, $v$ is the velocity component of $\alpha$, $r_{ij}$ is the relative distance between particles $i$ and $j$ in direction $\alpha$, and $f_{ij}$ is the force acting on particles $i$ and $j$ in direction $\beta$.

From the self-diffusion coefficient of the carrier ions $D_{i} \left( {i = + , - } \right)$ and N–E relation, the ionic conductivity was determined by:

14 $$\begin{array}{*{20}c} {D_{i} = \mathop {\lim }\limits_{t \to \infty } \frac{1}{6t}\langle\mathop \sum \limits_{i}^{N} \left| {r_{i} \left( t \right) - r_{i} \left( 0 \right)} \right|^{2}\rangle ,} \\ \end{array}$$

15 $$\begin{array}{*{20}c} {\sigma_{NE} = \frac{{e^{2} }}{{Vk_{B} T}}\left( {N_{ + } z_{ + }^{2} D_{ + } + N_{ - } z_{ - }^{2} D_{ - } } \right),} \\ \end{array}$$

or calculated using the E relation:

16 $$\begin{array}{*{20}c} {\sigma_{E} = \mathop {\lim }\limits_{t \to \infty } \frac{{e^{2} }}{{6tVk_{B} T}}\mathop \sum \limits_{i}^{N} z_{i} z_{j} \langle\left[ {r_{i} \left( t \right) - r_{i} \left( 0 \right)} \right]\left[ {r_{j} \left( t \right) - r_{j} \left( 0 \right)} \right]\rangle,} \\ \end{array}$$

where $r_{i} \left( t \right)$ is the coordinate of particle *i* at time *t*, $z_{i}$ is the charge of particle $i$, and $e$ is the elementary electric charge. The difference between the N–E and E relations is the treatment of the couplings between the ionic species, as discussed in previous studies^16,26^, where N–E corresponds to the limit of the uncorrelated motion between ionic species. In the experiments, similar arguments were made regarding the ratio of the conductivities determined from nuclear magnetic resonance measurements (corresponding to the N–E equation) and electrochemical impedance measurements (corresponding to the E equation). However, in the numerical calculations, the E equation was prone to the same uncertainty problem as the viscosity calculation, thereby requiring more samples than the N–E equation. Therefore, the evaluation is often based on the N–E equation, which has recently been considered for ion pairing^27^.

### Improved prediction of shear viscosity by conventional MD method

For an isotropic system, averaging over the three off-diagonal components of the stress tensor improves the statistical evaluation of shear viscosity. To gather better statistics, we adopted the following relation^28,29^:

17 $$\begin{array}{*{20}c} {\eta = \mathop {\lim }\limits_{t \to \infty } \frac{V}{{10k_{B} T}}\mathop \smallint \limits_{0}^{t} dt^{\prime}\langle P_{\alpha \beta }^{{{\text{OS}}}} \left( {t^{\prime}} \right)P_{\alpha \beta }^{{{\text{OS}}}} \left( 0 \right)\rangle, } \\ \end{array}$$

$P_{\alpha \beta }^{{{\text{OS}}}}$ is the symmetrized traceless portion of the stress tensor, defined as

18 $$\begin{array}{*{20}c} {P_{\alpha \beta }^{{{\text{OS}}}} = {\upomega }_{\alpha \beta } \left( {\frac{{P_{\alpha \beta } + P_{\beta \alpha } }}{2} - \frac{{\delta_{\alpha \beta } }}{3}\mathop \sum \limits_{\gamma } P_{\gamma \gamma } } \right),} \\ \end{array}$$

where $\delta_{\alpha \beta }$ is the Kronecker delta, and $\omega_{\alpha \beta }$ is the weighting factor, $\omega_{\alpha \beta } = 1 {\text{for }}\alpha \ne \beta$, and $\omega_{\alpha \beta } = 4/3\;{\text{for }}\alpha = \beta$. Notably, the factor of 10 in the denominator of Eq.﻿ (17) resulted from $\omega_{\alpha \beta }$ for six off-diagonal terms and three diagonal terms^28,29^.

The time integral in Eq. (17) was truncated within a certain simulation time. Additionally, integrating the slow decay of the autocorrelation function may cause numerical problems. To obtain the valid viscosity, the running integral over time was fitted to the following model function^25,30,31^:

19 $$\begin{array}{*{20}c} {h\left( t \right) = H\zeta \tau_{1} \left( {1 - {\text{exp}}\left( { - \frac{t}{{\tau_{1} }}} \right)} \right) + H\left( {1 - \zeta } \right)\tau_{2} \left( {1 - {\text{exp}}\left( { - \frac{t}{{\tau_{2} }}} \right)} \right),} \\ \end{array}$$

where H, *θ*,* τ*_1_, and τ_2_ are the fitting parameters. In this study, for the EC–EMC–LiPF_6_ case, the parameters were defined by integrating up to 400 ps. Afterward, the viscosity was calculated by extrapolating the model to infinite time: $\eta = \mathop {{\text{lim}}}\limits_{t \to \infty } h\left( t \right) \simeq H\zeta \tau_{1} + H\left( {1 - \zeta } \right)\tau_{2}$.

### Estimation of the degree of ion dissociation

To determine the degree of ion dissociation, α, we used the structural information of the MD trajectories. That is, the ratio of the number of associated cation–anion pairs to the total number of salts in a snapshot structure was analyzed and averaged over a trajectory to obtain the degree of association (1 − α). In this study, because the salts considered were LiPF_6_, LiFSA, and LiTFSA, the cation was limited to Li^+^. Therefore, the associated pairs were defined as anions around Li within a threshold distance. For simplicity, we chose the distance between Li and P atoms for the PF_6_ anion or Li and O atoms for the FSA and TFSA anions. The threshold values were set to 4.2 Å for LiPF_6_ and 2.8 Å for LiFSA and LiTFSA, which were determined using the radial distribution function of Li and the selected atom in the anion. Notably, this procedure is the same as that used for the EC–LiPF_6_ electrolytes by Kumar and Seminario^32^.

### Molecular radius estimation for salts

To estimate the molecular radii of LiPF_6_, LiFSA, and LiTFSA, we performed quantum chemical calculations using the Gaussian program^33^. After the geometry optimization of the salt structure with the B3LYP functional and the 6–31++G** basis sets, molecular volume (“Volume” keyword) was estimated. The ion radius was estimated from the obtained volume by assuming that the shape of the molecule was spherical.

### MD simulation setup

In this study, we performed two simulation procedures: a conventional MD evaluation and a fast MD evaluation. The former method was also adopted for the model-fitting simulation. The only difference between the two protocols was the time length of the MD run (described below).

The force field parameters and partial atomic charges for molecules, except oils, Li^+^, and PF_6_^−^, were adopted by the GAFF^23^ and the restricted electrostatic potential (RESP) charge^34^ using the Antechamber program^35^. The GAFF has a simple harmonic functional form with the Lennard–Jones and Coulomb potentials:

20 $$\begin{aligned} E_{{{\text{tot}}}} & = \mathop \sum \limits_{{{\text{bonds}}}} k_{r} \left( {r - r_{0} } \right)^{2} + \mathop \sum \limits_{{{\text{angles}}}} k_{\theta } \left( {\theta - \theta_{0} } \right)^{2} + \mathop \sum \limits_{{{\text{dihedrals}}}} \frac{{\nu_{n} }}{2}\left[ {1 + \cos \left( {n\phi - \gamma } \right)} \right] \\ & \quad + \mathop \sum \limits_{i < j} 4 \epsilon_{ij} \left[ {\left( {\frac{{\sigma_{ij} }}{{r_{ij} }}} \right)^{12} - \left( {\frac{{\sigma_{ij} }}{{r_{ij} }}} \right)^{6} } \right] + \mathop \sum \limits_{i < j} \frac{{q_{i} q_{j} }}{{r_{ij} }}, \\ \end{aligned}$$

where $E_{{{\text{tot}}}}$ is the total potential energy; $k_{r} , k_{\theta } , \nu_{n}$ are force constants; $r_{0}$ and $\theta_{0}$ are equilibrium bond length and angle, respectively; $n$ and $\gamma$ are the multiplicity and phase angle, respectively, for torsional angle parameters; $\epsilon_{ij}$ and $\sigma_{ij}$ are the Lennard–Jones energy and size parameters, respectively, for atom pairs of *i* and *j*; $r_{ij}$ is the distance between atom *i* and *j*; and $q_{i}$ is the partial charge of atom *i*. For the RESP calculation, we used information obtained from the Gaussian program^33^ with HF/6-31G//B3LYP/6–31++G** level. The oil molecules were adopted by the GAFF force field and AM1-BCC charges^36^ assigned by the Antechamber program. The force field parameters and charge for Li^+^ were obtained from Joung et al.^37^, whereas those of PF_6_^−^ were obtained from Canongia Lopes et al.^38^.

The initial coordinates for the electrolytes were generated using the Packmol program^39^. Periodic boundary conditions (PBCs) were applied to a cubic box. Cutoffs for Coulomb and van der Waals interactions were taken at 10 Å, and the particle–particle particle–mesh (PPPM) method with an accuracy of $8 \times 10^{ - 5}$ was used for long-range interactions.

All the MD simulations were performed using the LAMMPS package^40^. The initial configurations were relaxed using an energy minimization scheme and short-run (0.01 ns) in the constant-temperature, constant-volume (NVT) ensemble. Thereafter, an annealing process was used to accelerate the equilibration process. After being heated to approximately 1.8 times the desired temperature, the simulation box was equilibrated for 0.25 ns in the isobaric–isothermal (NPT) ensemble. The box was cooled to the target temperature, and afterward, the equilibration run was performed in the NPT ensemble for 0.7 and 2.0 ns for the fast and conventional MD runs, respectively. The average volume was computed over the final 0.2 ns, and the box size was changed to the average volume for subsequent runs. After further NVT ensemble for at least 0.5 ns, the production run was performed in the NVT ensemble for 1.0 and 20 ns for the fast and conventional MD runs, respectively. The time step and pressure were set as 1 fs and 1 atm, respectively. A Nosé–Hoover thermostat and barostat were used to control the temperature and pressure, respectively.

## Experimental details

### Materials

Lithium bis(fluorosulfonyl)amide (LiFSA, battery grade), fluoroethylene carbonate (FEC, battery grade) and dimethyl carbonate (DMC, battery grade) were obtained from Kishida Chemical. Acetonitrile (ACN, super dehydrated) was obtained from Wako Pure Chemical Industries. Methyl acetate (MA, anhydrous, 99.5%) was obtained from Sigma-Aldrich. Additionally, 1,1,2,2-Tetrafluoro-1-(2,2,2-trifluoroethoxy) ethane (D2, > 99%) was obtained from Tokyo Chemical Industry.

### Viscosity measurement

Viscosity measurements were performed using a rotational rheometer (HAKKE RehoStress 600) with a cone and plate sensor (60 mm diameter, 1° cone angle). The shear rate was between 10 and 600 1/s.

### Conductivity measurement

A coin cell (14 mm in diameter) was fabricated using lithium manganate oxide (LiMn_2_O_4_) as the cathode, lithium titanate (Li_2_TiO_3_) as the anode, and a filter paper impregnated with the target electrolyte. The alternating current (AC) impedance spectra of the cell were measured in the frequency range from 7 MHz to 20 mHz using a BioLogic SP-300 potentiostat. By fitting the Nyquist plot obtained from the spectra to the equivalent circuit (see Supplementary Information), the ionic conductivity was estimated from the solution resistance using a surface area of 1.5386 cm^2^, a thickness of 0.356 mm, and porosity of 100%.

## Supplementary Information

Supplementary Information.
