# Supplementary material for: Fast evaluation technique for the shear viscosity and ionic conductivity of electrolyte solutions
Source: Sci Rep. 2022 May 4;12:7291. doi: 10.1038/s41598-022-10704-z (PMC9068762; doi:10.1038/s41598-022-10704-z)
Supplement: Supplementary file 1 — Supplementary Information. [file 41598_2022_10704_MOESM1_ESM.pdf]

Fast evaluation technique for the shear viscosity and ionic conductivity of electrolyte solution

Takeshi Baba, Seiji Kajita, Tohru Shiga, and Nobuko Ohba

Toyota Central R&D Labs., Inc., 41-1, Yokomichi, Nagakute, Aichi 480-1192, Japan

Supplementary information

S1 Estimation of boiling point for $LiPF_6$, LiFSA, LiTFSA

The proposed estimating model for shear viscosity included the boiling temperature (T_b) term. For electrolyte solutions, we assumed that the boiling point of the solution is determined by the boiling points of the components and their mole fractions. However, to our knowledge, the boiling point of the supporting salts, $LiPF_6$, LiFSA, and LiTFSA, were not reported. We focus on the melting point of those salts were reported and both melting and boiling point were known for several Li-salts. A regression model for the melting and boiling points was created from Li-salts, in which the both points were known (See Table S1), and the boiling points of $LiPF_6$, LiFSA, and LiTFSA were estimated from their melting points using the regression model.

Table S1 Melting and boiling points of the reference Li-salts.

Li-Salts	Melting point [°C]	Boiling point [°C]	Reference
LiF	848	1671	S1
LiCl	613	1360	S1
LiBr	552	1265	S2
LiI	469	1171	S2
Li_2CO_3	723	1310	S2
LiOH	462	924	S2
Li_2O	1570	2563	S3
$LiPF_6$	190	–	S4
LiFSA	145	–	S5
LiTFSA	236	–	S6

S2 Properties of the reference molecules

Table S2, S3, and S4 were indicated the data of reference molecules to use the fitting for fast evaluation model.

Table S2 Boiling point and shear viscosity of the reference organics.

Molecular name	Abbreviation	Chemical formula	Boiling point [°C]	Viscosity (25°C) [mPa·s]	Reference
Ethylene carbonate	EC	$C_3H_4O_3$	248	2.263	S1, S7
Propylene carbonate	PC	$C_4H_6O_3$	241.7	2.512	S1, S8
Butylene carbonate	BC	$C_5H_8O_3$	240	3.2	S9, S10
Dimethyl carbonate	DMC	$C_3H_6O_3$	90.5	0.5771	S1, S11
Ethyl methyl carbonate	EMC	$C_4H_8O_3$	110	0.65	S9, S12
Diethyl carbonate	DEC	$C_5H_{10}O_3$	126	0.746	S1, S13
γ-Butyrolactone	GBL	$C_4H_6O_2$	204	1.738	S9, S14
γ-Valerolactone	GVL	$C_5H_8O_2$	208	1.86	S1, S15
Acetonitrile	AN	C_2H_3N	81.6	0.3443	S1, S16
Glutaronitrile	GLN	$C_5H_6N_2$	286	5.3	S1, S10
Adipodinitrile	ADN	$C_6H_8N_2$	295	6	S1, S10
Methoxyacetonitrile	MAN	C_3H_5NO	119	0.7	S1, S10
3-Methoxypropionitrile	MPN	C_4H_7NO	163	1.1	S1, S10
Dimethyl formamide	DMF	C_3H_7NO	152.8	0.8135	S1, S17
Dimethyl acetamide	DMA	C_4H_9NO	165	0.919	S1, S18
N-Methyloxazolidone	NMO	$C_4H_7NO_2$	270	2.5	S10
N,N’-Dimethylimidazolidinone	DMI	$C_5H_{10}N_2O$	221	1.9	S1, S10
N-Methylpyrrolidone	NMP	C_5H_9NO	202	1.666	S1, S19
Nitromethane	NM	CH_3NO_2	101.1	0.615	S1, S20
Nitroethane	NE	$C_2H_5NO_2$	114.1	0.661	S1, S21
Sulfolane	SL	$C_4H_8O_2S$	285	10.3	S1, S22
Dimethyl sulfoxide	DMSO	C_2H_6OS	189	1.996	S1, S23
Trimethyl phosphate	TMP	$C_3H_9O_4P$	197.2	2.032	S1, S24
1,2-Dimethoxyethane	DME	$C_4H_{10}O_2$	85	0.417	S1, S25
Diglyme	DG	$C_6H_{14}O_3$	162	1.003	S1, S26
Triglyme	TG	$C_8H_{18}O_4$	216	2.009	S1, S26
Tetraglyme	TeG	$C_{10}H_{22}O_5$	275.3	3.294	S1, S26
1,2-Diethoxyethane	DEE	$C_6H_{14}O_2$	121.4	0.593	S1, S25
1,3-Dioxolane	DOx	$C_3H_6O_2$	78	0.5886	S1, S27

Molecular name	Abbreviation	Chemical formula	Boiling point [°C]	Viscosity (25°C) [mPa·s]	Reference
Tetrahydrofuran	THF	C_4H_8O	66	0.4631	S1, S16
2-Methyltetrahydrofuran	2-MeTHF	$C_5H_{10}O$	78	0.4776	S1, S28
4-Methyl-1,3-dioxolane	4-MeDOL	$C_4H_8O_2$	85	0.6	S29
Methanol	MeOH	CH_4O	64.7	0.55	S1, S13
Toluene		C_7H_8	110.6	0.554	S1, S13
Chloroform		$CHCl_3$	61.12	0.545	S1, S30
Acetone		C_3H_6O	56.08	0.3029	S1, S31
Ethylenediamine	EDA	$C_2H_8N_2$	117	1.4	S1, S32
Ethyl acetate	EA	$C_4H_8O_2$	77.1	0.43	S1, S33
Ethyl propanoate	EP	$C_5H_{10}O_2$	99.2	0.492	S1, S34
Methyl formate	MF	$C_2H_4O_2$	31.5	0.328	S1, S35
Methyl acetate	MA	$C_3H_6O_2$	56.7	0.367	S1, S33
Methyl propionate	MP	$C_4H_8O_2$	79.8	0.434	S1, S36
Methyl butyrate	MB	$C_5H_{10}O_2$	102.8	0.539	S1, S36
Acetic acid	AA	$C_2H_4O_2$	118.1	1.115	S1, S37

Table S3 Shear viscosity of the reference oil molecules. All data were taken from the previous study[S38].

Molecular name	Abbreviation	Chemical Formula	Viscosity (40°C) [mPa·s]
13-n-Dodecylhexacosane	13nddh	$C_{38}H_{78}$	19.04
9-n-Hexylheptadecane	9nhhd	$C_{23}H_{48}$	5.56
9-Cyclohexylheptadecane	9chhd	$C_{23}H_{46}$	9.05
11-Cyclohexylheneicosane	11chh	$C_{27}H_{54}$	13.68
1,1-Dicyclohexyltetradecane	1_1dchtd	$C_{26}H_{50}$	29.52
13-Phenylpentacosane	13php	$C_{31}H_{56}$	17.63
11-Decyldocosane	11ndd	$C_{31}H_{64}$	12.99
1,1-Diphenyltetradecane	1_1dphtd	$C_{26}H_{38}$	15.35
diisooctyl sebacate	diiso_seb	$C_{25}H_{48}O_4$	11.90
2-methoxy-4-octadecyliminomethyl-phenol	2m4odp	$C_{26}H_{45}NO_2$	34.07
1-cyclohexyl-2-(cyclohexylmethyl)heptadecane	1c2cmh	$C_{30}H_{58}$	24.30
13-Cyclohexylpentacosane	13cp	$C_{31}H_{62}$	18.99

Table S4 The properties of the reference electrolytes. Asterisk mark indicates the data set for model fitting.

Electrolytes	Temperature [K]	Shear viscosity [mPa·s]	Ionic conductivity [mS/cm]	Data for viscosity model (eq.2)	Data for conductivity model (eq.5)
PC-$LiPF_6$ (100:1 molar ratio) [S39]	298.15	2.77	2.1	*	*
PC-$LiPF_6$ (20:1 molar ratio) [S39]	298.15	4.69	5.8	*	*
PC-$LiPF_6$ (11:1 molar ratio) [S39]	298.15	8.03	6.0	*	*
PC-$LiPF_6$ (10:1 molar ratio) [S39]	298.15	9.93	5.8	*	*
PC-$LiPF_6$ (6.7:1 molar ratio) [S39]	298.15	18.6	4.1	*	*
PC-$LiPF_6$ (5:1 molar ratio) [S39]	298.15	46.9	2.3	*	*
PC-$LiPF_6$ (4:1 molar ratio) [S39]	298.15	120	1.2	*	*
PC-$LiPF_6$ (3.3:1 molar ratio) [S39]	298.15	223	0.61	*	*
PC-$LiPF_6$ (2.85:1 molar ratio) [S39]	298.15	389	0.43	*	*
DMC-LiFSA (10 mol%) [S40]					
	283.15	2.567	7.70	*	*
	293.15	2.099	8.90	*	*
	313.15	1.483	11.11	*	*
	333.15	1.112	13.19	*	*
DMC-LiFSA (20 mol%) [S40]					
	283.15	10.571	6.69	*	*
	293.15	7.769	8.38	*	*
	313.15	4.664	11.69	*	*
	333.15	3.163	15.33	*	*
DMC-LiFSA (25 mol%) [S40]					
	283.15	22.539	–	*	
	293.15	15.408	–	*	
	313.15	8.444	–	*	
	333.15	5.344	–	*	

Electrolytes	Temperature [K]	Shear viscosity [mPa·s]	Ionic conductivity [mS/cm]	Data for viscosity model (eq.2)	Data for conductivity model (eq.5)
DMC-LiFSA (30 mol%) [S40]					
	283.15	47.954	–	*	
	293.15	27.250	–	*	
	313.15	15.285	–	*	
	333.15	9.027	–	*	
DMC-LiFSA (35 mol%) [S40]					
	283.15	101.83	–	*	
	293.15	60.679	–	*	
	313.15	27.250	–	*	
	333.15	14.951	–	*	
DMC-LiFSA (40 mol%) [S40]					
	283.15	228.60	–	*	
	293.15	129.17	–	*	
	313.15	50.214	–	*	
	333.15	25.119	–	*	
DMC-LiFSA (45 mol%) [S40]					
	283.15	560.81	–	*	
	293.15	279.24	–	*	
	313.15	95.880	–	*	
	333.15	43.863	–	*	
EC-LiFSA (5 mol%) [S40]					
	303.15	4.428	–	*	
	313.15	3.659	8.75	*	*
	323.15	2.996	10.22	*	*
	333.15	2.537	11.73	*	*
EC-LiFSA (10 mol%) [S40]					
	303.15	9.715	–	*	
	313.15	7.086	11.15	*	*
	323.15	5.610	13.27	*	*
	333.15	4.740	15.45	*	*

Electrolytes	Temperature [K]	Shear viscosity [mPa·s]	Ionic conductivity [mS/cm]	Data for viscosity model (eq.2)	Data for conductivity model (eq.5)
EC-LiFSA (20 mol%) [S40]					
	303.15	47.269	–	*	
	313.15	30.605	–	*	
	323.15	21.148	–	*	
	333.15	15.290	–	*	
EC-LiFSA (25 mol%) [S40]					
	303.15	94.365	–	*	
	313.15	56.674	–	*	
	323.15	37.692	–	*	
	333.15	26.166	–	*	
EC-LiFSA (30 mol%) [S40]					
	303.15	162.47	–	*	
	313.15	93.946	–	*	
	323.15	59.116	–	*	
	333.15	39.634	–	*	
EC-LiFSA (35 mol%) [S40]					
	303.15	261.85	–	*	
	313.15	145.37	–	*	
	323.15	88.380	–	*	
	333.15	57.895	–	*	
EC-LiFSA (40 mol%) [S40]					
	303.15	431.46	–	*	
	313.15	229.94	–	*	
	323.15	133.77	–	*	
	333.15	92.133	–	*	
EC-LiFSA (45 mol%) [S40]					
	303.15	742.16	–	*	
	313.15	370.31	–	*	
	323.15	206.98	–	*	
	333.15	126.28	–	*	

Electrolytes	Temperature [K]	Shear viscosity [mPa·s]	Ionic conductivity [mS/cm]	Data for viscosity model (eq.2)	Data for conductivity model (eq.5)
PC-LiFSA (10 mol%) [S40]					
	273.15	23.269	2.80	*	*
	293.15	10.999	5.39	*	*
	313.15	6.336	8.59	*	*
	333.15	4.120	12.21	*	*
PC-LiFSA (20 mol%) [S40]					
	273.15	187.28	–	*	
	293.15	57.095	–	*	
	313.15	24.616	–	*	
	333.15	13.090	–	*	
PC-LiFSA (25 mol%) [S40]					
	273.15	467.03	–	*	
	293.15	120.60	–	*	
	313.15	45.689	–	*	
	333.15	21.766	–	*	
PC-LiFSA (30 mol%) [S40]					
	273.15	948.193	–	*	
	293.15	217.08	–	*	
	313.15	75.211	–	*	
	333.15	34.387	–	*	
PC-LiFSA (35 mol%) [S40]					
	273.15	1875.1	–	*	
	293.15	370.58	–	*	
	313.15	118.26	–	*	
	333.15	50.891	–	*	
PC-LiFSA (40 mol%) [S40]					
	273.15	3770.7	–	*	
	293.15	639.65	–	*	
	313.15	185.77	–	*	
	333.15	74.466	–	*	

Electrolytes	Temperature [K]	Shear viscosity [mPa·s]	Ionic conductivity [mS/cm]	Data for viscosity model (eq.2)	Data for conductivity model (eq.5)
PC-LiFSA (45 mol%) [S40]					
	273.15	8162.5	–	*	
	293.15	1129.0	–	*	
	313.15	296.47	–	*	
	333.15	110.46	–	*	
MP-LiTFSA (2:1 molar ratio) [S41]	298.15	21.6	2.3	*	*
DMC-LiTFSA (2:1 molar ratio) [S41]	298.15	53.7	1.2	*	*
BC-LiTFSA (2:1 molar ratio) [S41]	298.15	323	0.2	*	*
BC-DMC(1:3)-LiTFSA (2:1 molar ratio) [S41]	298.15	94.444	–	*	
BC-DMC(1:1)-LiTFSA (2:1 molar ratio) [S41]	298.15	157.578	–	*	
DMC-MP(1:1)-LiTFSA (2:1 molar ratio) [S41]	298.15	38.226	–	*	
GLN- LiTFSA (1 M) [S42]	293.15	20.70	1.99	*	*
	333.15	6.49	5.51	*	*
MGLN-LiTFSA (1 M) [S42]	293.15	23.08	1.48	*	*
	333.15	6.30	4.93	*	*
EC-LiTFSA (2:1 molar ratio) [S43]					
	293.15	2332	0.120	*	*
	303.15	888.7	0.269	*	*
	313.15	401.7	0.521	*	*
	323.15	206.9	0.892	*	*
	333.15	119.7	1.40	*	*
	343.15	76.69	2.04	*	*
	353.15	50.29	2.83	*	*
EC-LiTFSA (6:1 molar ratio) [S43]					
	283.15	47.35	2.43	*	*
	293.15	29.51	3.63	*	*

Electrolytes	Temperature [K]	Shear viscosity [mPa·s]	Ionic conductivity [mS/cm]	Data for viscosity model (eq.2)	Data for conductivity model (eq.5)
	303.15	19.94	5.07	*	*
	313.15	14.30	6.71	*	*
	323.15	10.71	8.57	*	*
	333.15	8.299	10.6	*	*
	343.15	6.613	12.7	*	*
	353.15	5.381	14.9	*	*
PC-LiTFSA (6:1 molar ratio) [S43]					
	283.15	33.87	2.15	*	*
	293.15	21.90	3.03	*	*
	303.15	15.11	4.04	*	*
	313.15	10.94	5.01	*	*
	323.15	8.271	6.21	*	*
	333.15	6.448	7.07	*	*
	343.15	5.175	7.94	*	*
	353.15	4.229	8.96	*	*
EC-DEC (50:50 vol%)-LiTFSA (1 M) [S43]					
	283.15	7.314	3.36	*	*
	293.15	5.531	4.26	*	*
	303.15	4.318	5.23	*	*
	313.15	3.478	6.30	*	*
	323.15	2.874	7.43	*	*
	333.15	2.423	8.60	*	*
	343.15	2.071	9.82	*	*
	353.15	1.823	11.1	*	*
EC-EMC-MA (30:40:30 wt%)-$LiPF_6$ (0.5 m)[S44]					
	283.15	1.74	8.62		*
	293.15	1.32	9.97		*
	303.15	1.14	11.15		*
	313.15	1.00	12.07		*
EC-EMC-MA (30:50:20 wt%)-$LiPF_6$ (0.5 m)[S44]					
	283.15	1.95	7.30		*

Electrolytes	Temperature [K]	Shear viscosity [mPa·s]	Ionic conductivity [mS/cm]	Data for viscosity model (eq.2)	Data for conductivity model (eq.5)
	293.15	1.52	8.70		*
	303.15	1.30	9.93		*
	313.15	1.00	10.73		*
EC-EMC-MA (30:60:10 wt%)-$LiPF_6$ (0.5 m)[S44]					
	283.15	2.22	7.08		*
	293.15	1.73	8.31		*
	303.15	1.43	9.49		*
	313.15	1.23	10.39		*
EC-EMC-MA (30:70 wt%)-$LiPF_6$ (0.5 m)[S44]					
	283.15	2.33	5.77		*
	293.15	1.91	6.84		*
	303.15	1.60	7.97		*
	313.15	1.36	9.05		*
EC-EMC-MA (30:40:30 wt%)-$LiPF_6$ (1.0 m)[S44]					
	283.15	2.88	11.53		*
	293.15	2.15	13.64		*
	303.15	1.80	15.41		*
	313.15	1.47	16.95		*
EC-EMC-MA (30:50:20 wt%)-$LiPF_6$ (1.0 m)[S44]					
	283.15	3.38	9.19		*
	293.15	2.38	11.05		*
	303.15	1.97	12.49		*
	313.15	1.67	14.19		*
EC-EMC-MA (30:60:10 wt%)-$LiPF_6$ (1.0 m)[S44]					
	283.15	3.98	7.91		*
	293.15	2.82	9.58		*
	303.15	2.31	11.24		*
	313.15	1.94	12.44		*

Electrolytes	Temperature [K]	Shear viscosity [mPa·s]	Ionic conductivity [mS/cm]	Data for viscosity model (eq.2)	Data for conductivity model (eq.5)
EC-EMC-MA (30:70 wt%)-$LiPF_6$ (1.0 m)[S44]					
	283.15	3.88	6.44		*
	293.15	3.05	7.94		*
	303.15	2.47	9.57		*
	313.15	2.06	11.05		*
EC-EMC-MA (30:40:30 wt%)-$LiPF_6$ (1.5 m)[S44]					
	283.15	4.83	10.39		*
	293.15	3.63	12.66		*
	303.15	2.90	15.02		*
	313.15	2.41	16.74		*
EC-EMC-MA (30:50:20 wt%)-$LiPF_6$ (1.5 m)[S44]					
	283.15	5.79	8.53		*
	293.15	3.81	10.64		*
	303.15	3.06	12.7		*
	313.15	2.50	14.7		*
EC-EMC-MA (30:60:10 wt%)-$LiPF_6$ (1.5 m)[S44]					
	283.15	6.87	7.03		*
	293.15	4.94	8.87		*
	303.15	3.71	10.8		*
	313.15	3.10	12.56		*
EC-EMC-MA (30:70 wt%)-$LiPF_6$ (1.5 m)[S44]					
	283.15	8.73	5.51		*
	293.15	6.25	7.10		*
	303.15	4.75	8.65		*
	313.15	3.75	10.14		*
EC-EMC-MA (30:40:30 wt%)-$LiPF_6$ (2.0 m)[S44]					
	283.15	7.59	9.15		*

Electrolytes	Temperature [K]	Shear viscosity [mPa·s]	Ionic conductivity [mS/cm]	Data for viscosity model (eq.2)	Data for conductivity model (eq.5)
	293.15	6.06	11.33		*
	303.15	4.69	13.35		*
	313.15	3.70	15.03		*
EC-EMC-MA (30:50:20 wt%)-$LiPF_6$ (2.0 m)[S44]					
	283.15	9.38	7.38		*
	293.15	7.33	9.35		*
	303.15	5.52	11.38		*
	313.15	4.29	13.3		*
EC-EMC-MA (30:60:10 wt%)-$LiPF_6$ (2.0 m)[S44]					
	283.15	11.79	6.39		*
	293.15	8.57	8.31		*
	303.15	6.25	10.29		*
	313.15	5.03	12.19		*
EC-EMC-MA (30:70 wt%)-$LiPF_6$ (2.0 m)[S44]					
	283.15	16.58	4.57		*
	293.15	11.40	6.17		*
	303.15	8.18	7.95		*
	313.15	6.25	9.75		*
EC-DMC-MA (30:40:30 wt%)-$LiPF_6$ (0.5 m)[S44]					
	283.15	1.50	9.94		*
	293.15	1.27	11.45		*
	303.15	1.09	12.83		*
	313.15	0.95	13.68		*
EC-DMC-MA (30:50:20 wt%)-$LiPF_6$ (0.5 m)[S44]					
	283.15	1.71	8.68		*
	293.15	1.41	10.15		*
	303.15	1.21	11.49		*
	313.15	1.05	12.44		*

Electrolytes	Temperature [K]	Shear viscosity [mPa·s]	Ionic conductivity [mS/cm]	Data for viscosity model (eq.2)	Data for conductivity model (eq.5)
EC-DMC-MA (30:60:10 wt%)-$LiPF_6$ (0.5 m)[S44]					
	283.15	1.78	8.32		*
	293.15	1.47	9.68		*
	303.15	1.25	10.85		*
	313.15	1.08	11.71		*
EC-DMC-MA (30:70 wt%)-$LiPF_6$ (0.5 m)[S44]					
	283.15	2.00	7.69		*
	293.15	1.64	9.38		*
	303.15	1.38	10.89		*
	313.15	1.18	12.38		*
EC-DMC-MA (30:40:30 wt%)-$LiPF_6$ (1.0 m)[S44]					
	283.15	2.37	12.46		*
	293.15	1.98	14.63		*
	303.15	1.64	16.71		*
	313.15	1.40	18.46		*
EC-DMC-MA (30:50:20 wt%)-$LiPF_6$ (1.0 m)[S44]					
	283.15	2.84	11.21		*
	293.15	2.26	13.37		*
	303.15	1.84	15.66		*
	313.15	1.55	17.06		*
EC-DMC-MA (30:60:10 wt%)-$LiPF_6$ (1.0 m)[S44]					
	283.15	3.16	10.20		*
	293.15	2.51	12.38		*
	303.15	2.06	14.63		*
	313.15	1.73	16.10		*
EC-DMC-MA (30:70 wt%)-$LiPF_6$ (1.0 m)[S44]					
	283.15	3.63	9.05		*

Electrolytes	Temperature [K]	Shear viscosity [mPa·s]	Ionic conductivity [mS/cm]	Data for viscosity model (eq.2)	Data for conductivity model (eq.5)
	293.15	2.85	11.14		*
	303.15	2.30	13.25		*
	313.15	1.92	14.97		*
EC-DMC-MA (30:40:30 wt%)-$LiPF_6$ (1.5 m)[S44]					
	283.15	3.81	12.77		*
	293.15	3.28	15.31		*
	303.15	2.62	17.99		*
	313.15	2.18	20.24		*
EC-DMC-MA (30:50:20 wt%)-$LiPF_6$ (1.5 m)[S44]					
	283.15	4.87	11.05		*
	293.15	3.77	13.55		*
	303.15	2.98	16.32		*
	313.15	2.43	18.74		*
EC-DMC-MA (30:60:10 wt%)-$LiPF_6$ (1.5 m)[S44]					
	283.15	5.93	9.30		*
	293.15	4.49	11.75		*
	303.15	3.50	14.20		*
	313.15	2.81	16.26		*
EC-DMC-MA (30:70 wt%)-$LiPF_6$ (1.5 m)[S44]					
	283.15	7.06	8.43		*
	293.15	5.17	10.60		*
	303.15	3.63	12.90		*
	313.15	3.21	15.35		*
EC-DMC-MA (30:40:30 wt%)-$LiPF_6$ (2.0 m)[S44]					
	283.15	6.90	11.03		*
	293.15	5.12	13.81		*
	303.15	4.02	16.51		*
	313.15	3.18	18.87		*

Electrolytes	Temperature [K]	Shear viscosity [mPa·s]	Ionic conductivity [mS/cm]	Data for viscosity model (eq.2)	Data for conductivity model (eq.5)
EC-DMC-MA (30:50:20 wt%)-$LiPF_6$ (2.0 m)[S44]					
	283.15	8.34	10.28		*
	293.15	6.10	12.88		*
	303.15	4.61	15.36		*
	313.15	3.56	17.68		*
EC-DMC-MA (30:60:10 wt%)-$LiPF_6$ (2.0 m)[S44]					
	283.15	10.19	8.40		*
	293.15	7.16	10.86		*
	303.15	5.33	13.43		*
	313.15	4.13	15.85		*
EC-DMC-MA (30:70 wt%)-$LiPF_6$ (2.0 m)[S44]					
	283.15	13.41	7.06		*
	293.15	9.29	9.38		*
	303.15	6.61	11.80		*
	313.15	5.10	13.93		*
EC-EMC (30:70 vol%)-LiFSA (1.0 M)[S45]	298.15	2.96	9.73		*
EC-EMC (30:70 vol%)-$LiPF_6$ (1.0 M)[S45]	298.15	3.00	9.33		*
EC-EMC (30:70 vol%)-LiTFSA (1.0 M)[S45]	298.15	3.40	7.57		*
EC-DMC (1:1 molar ratio)-$LiPF_6$ (0.771 m)[S46]	298.15	3.9	10.7		*
FEC-$LiPF_6$ (0.75 m)[S46]	298.15	12.3	2.9		*
EC-FEC (1:1 molar ratio)-$LiPF_6$ (0.75 m)[S46]	298.15	11.1	4.0		*
PC-FEC (1:1 molar ratio)-$LiPF_6$ (0.75 m)[S46]	298.15	11.2	3.5		*

Electrolytes	Temperature [K]	Shear viscosity [mPa·s]	Ionic conductivity [mS/cm]	Data for viscosity model (eq.2)	Data for conductivity model (eq.5)
BC-FEC (1:1 molar ratio)-$LiPF_6$ (0.75 m)[S46]	298.15	12.0	2.8		*
DBC-FEC (1:1 molar ratio)-$LiPF_6$ (0.75 m)[S46]	298.15	37.1	0.4		*
DPrC-FEC (1:1 molar ratio)-$LiPF_6$ (0.75 m)[S46]	298.15	8.4	2.5		*

MGLN: 2-Methylglutaronitrile, FEC: fluoroethylene carbonate, DBC: dibenzyl carbonate, DPrC: dipropyl carbonate

S3 Equivalent circuit model

Figure S1 shows the equivalent circuit used in the experimental analysis for ionic conductivity of electrolytes. The R_{e}, R_{SEI}, andR_{ct} parameters are the resistance of bulk, interfacial layer, and charge transfer, respectively. C_{SEI} and C_{dl} are the capacitance of interfacial layer and double-layer. Z_{w} is the Warburg impedance.

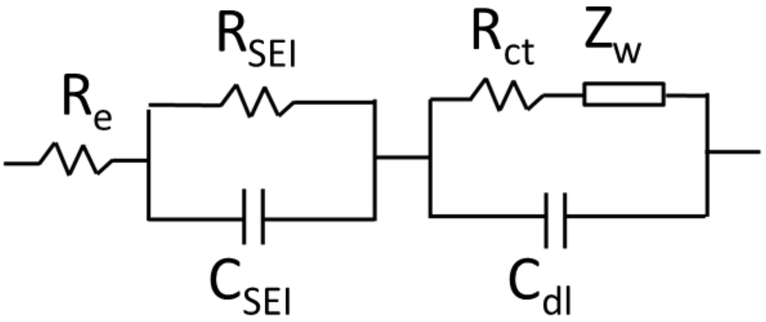

Fig. S1 Equivalent circuit model.

References

S1. Kim, S. *et al. Nucleic Acids Res.* **47**, D1102–D1109 (2019).

S2. Pence, H. E. & Williams, A. *J. Chem. Educ.* **87**, 1123–1124 (2010).

S3. Chase, M. W. *et al. J. Phys. Chem. Ref. Data* **4**, 1–176 (1975).

S4. Zinigrad, E. *et al. Thermochim. Acta* **438**, 184–191 (2005).

S5. Han, H.-B. *et al. J. Power Sources* **196**, 3623–3632 (2011).

S6. Dominey, L. A., Koch, V. R. & Blakley, T. J. *Electrochim. Acta* **37**, 1551–1554 (1992).

S7. Naejus, R. *et al. J. Chem. Thermodyn.* **29**, 1503–1515 (1997).

S8. Barthel, J., Neueder, R. & Roch, H. *J. Chem. Eng. Data* **45**, 1007–1011 (2000).

S9. Xu, K. *Chem. Rev.* **104**, 4303–4418 (2004).

S10. Ue, M., Ida, K. & Mori, S. *J. Electrochem. Soc.* **141**, 2989 (1994).

S11. Yang, C., Xu, W. & Ma, P. *J. Chem. Eng. Data* **49**, 1802–1808 (2004).

S12. Barthel, J. & Gores, H. J. in *Handbook of battery materials* 457–497 (John Wiley & Sons, Ltd, 1998).

S13. Yang, C. *et al. J. Chem. Eng. Data* **51**, 584–589 (2006).

S14. Ramkumar, D. H. & Kudchadker, A. P. *J. Chem. Eng. Data* **34**, 463–465 (1989).

S15. Aparicio, S. & Alcalde, R. *Phys. Chem. Chem. Phys.* **11**, 6455–6467 (2009).

S16. Das, M. & Roy, M. N. *J. Chem. Eng. Data* **51**, 2225–2232 (2006).

S17. Corradini, F. *et al. Bull. Chem. Soc. Jpn.* **68**, 1867–1872 (1995).

S18. Gill, D. S., Kumari, N. & Chauhan, M. S. *J. Chem. Soc., Faraday Trans. 1* **81**, 687–693 (1985).

S19. Langan, J. R. & Salmon, G. A. *J. Chem. Eng. Data* **32**, 420–422 (1987).

S20. Tu, C.-H., Lee, S.-L. & Peng, I.-H. *J. Chem. Eng. Data* **46**, 151–155 (2001).

S21. Holcomb, D. & Dorsey, C. L. *Ind. Eng. Chem.* **41**, 2788–2792 (1949).

S22. Ponomarenko, S. *et al. Zh. Obshch. Khim.* **65**, 190–198 (1995).

S23. Chauhan, M. S. *et al. Collect. Czech. Chem. Commun.* **60**, 43–54 (1995).

S24. Kannan, S. & Kishore, K. *J. Chem. Eng. Data* **44**, 649–655 (1999).

S25. Ku, H.-C. & Tu, C.-H. *J. Chem. Eng. Data* **50**, 608–615 (2005).

S26. Pal, A. & Singh, Y. P. *J. Chem. Eng. Data* **41**, 1008–1011 (1996).

S27. Gascón, I. *et al. J. Chem. Eng. Data* **50**, 722–726 (2005).

S28. Vallés, C. *et al. J. Chem. Eng. Data* **51**, 1105–1109 (2006).

S29. *Denkikagakubinran* (ed. The Electrochemical Society of Japan) 313–318 (Maruzen Publishing, 2013).

S30. Sovilj, M. N. *J. Chem. Eng. Data* **40**, 1058–1061 (1995).

S31. Hafez, M. & Hartland, S. *J. Chem. Eng. Data* **21**, 179–182 (1976).

S32. Fialkov, Yu. Ya., Chviruk, O. V. & Kudra, O. K. *Zh. Obshch. Khim.* **35**, 1523 (1965).

S33. Wankhede, N. *et al. J. Chem. Thermodyn.* **38**, 1664–1668 (2006).

S34. Djojoputro, H. & Ismadji, S. *J. Chem. Eng. Data* **50**, 727–731 (2005).

S35. Lu, H. *et al. J. Chem. Eng. Data* **46**, 631–634 (2001).

S36. Mariano, A. *et al. Phys. Chem. Liq.* **49**, 720–728 (2011).

S37. Gonzalez, B., Dominguez, A. & Tojo, J. *J. Chem. Eng. Data* **49**, 1590–1596 (2004).

S38. Kajita, S., Kinjo, T. & Nishi, T. *Commun. Phys.* **3**, 1–11 (2020).

S39. Kondo, K. *et al. J. Phys. Chem. B* **104**, 5040–5044 (2000).

S40. Neuhaus, J., Harbou, E. von & Hasse, H. *J. Power Sources* **394**, 148–159 (2018).

S41. Galle Kankanamge, S. R. & Kuroda, D. G. *J. Phys. Chem. B* **124**, 1965–1977 (2020).

S42. Farhat, D. *et al. J. Electrochem. Soc.* **166**, A3487 (2019).

S43. Nilsson, V. *et al. ACS Appl. Energy Mater.* **3**, 200–207 (2020).

S44. Logan, E. R. *et al. J. Electrochem. Soc.* **165**, A21 (2018).

S45. Han, H.-B. *et al. J. Power Sources* **196**, 3623–3632 (2011).

S46. Wang, Z., Hofmann, A. & Hanemann, T. *Electrochim. Acta* **298**, 960–972 (2019).
